# From Pathophysiology to Practice: Evolving Pharmacological Therapies, Clinical Complications, and Pharmacogenetic Considerations in Portal Hypertension

**DOI:** 10.3390/metabo15020072

**Published:** 2025-01-23

**Authors:** Michał Porada, Łukasz Bułdak

**Affiliations:** 1 Students’ Scientific Society, Department of Internal Medicine and Clinical Pharmacology, Medical University of Silesia, Medyków 18, 40-752 Katowice, Poland; s86115@365.sum.edu.pl; 2 Department of Internal Medicine and Clinical Pharmacology, Medical University of Silesia, Medyków 18, 40-752 Katowice, Poland

**Keywords:** portal hypertension, pharmacological treatments, hepatic circulation, beta-blockers, liver cirrhosis

## Abstract

**Background:** Portal hypertension is a major complication of chronic liver diseases, leading to serious issues such as esophageal variceal bleeding. The increase in portal vein pressure is driven by both an organic component and a functional component, including tonic contraction of hepatic stellate cells. These processes result in a pathological rise in intrahepatic vascular resistance, stemming from partial impairment of hepatic microcirculation, which is further exacerbated by abnormalities in extrahepatic vessels, including increased portal blood flow. **Objectives:** This review aims to provide a comprehensive overview of the evolving pharmacological therapies for portal hypertension, with consideration and discussion of pathophysiological mechanisms, clinical complications, and pharmacogenetic considerations, highlighting potential directions for future research. **Methods:** A review of recent literature was performed to evaluate current knowledge and potential therapeutic strategies in portal hypertension. **Results:** For over 35 years, non-selective beta-blockers have been the cornerstone therapy for portal hypertension by reducing portal vein inflow as an extrahepatic target, effectively preventing decompensation and variceal hemorrhages. However, since not all patients exhibit an adequate response to non-selective beta-blockers (NSBBs), and some may not tolerate NSBBs, alternative or adjunctive therapies that enhance the effects of NSBBs on portal pressure are being investigated in preclinical and early clinical studies. **Conclusions:** A better understanding of pharmacogenetic factors and pathophysiological mechanisms could lead to more individualized and effective treatments for portal hypertension. These insights highlight potential directions for future research.

## 1. Introduction

Portal hypertension (PH) is a clinical syndrome characterized by elevated blood pressure within the portal venous system. This condition is the primary underlying factor contributing to a range of significant complications, including ascites, jaundice, variceal hemorrhage, and an elevated risk of spontaneous bacterial peritonitis or other bacterial infections. Additionally, portal hypertension is associated with hepatic encephalopathy, hepatorenal syndrome, and hepatic failure [1]. The primary cause is chronic liver disease (CLD), which may be attributable to a range of diverse etiological factors and generally arises as a result of prolonged injury, leading to excessive deposition of extracellular matrix (ECM) and impairment of microvascular function [2]. Prolonged activation of the wound-healing response can result in the progressive replacement of hepatic parenchyma with fibrotic scar tissue [3]. The alterations arising from tissue remodeling in the cirrhotic liver are correlated with elevated intrahepatic resistance to portal blood flow, which consequently results in an increase in portal venous pressure [4,5]. Consequently, blood is redirected to the systemic circulation through collateral pathways that circumvent the liver. The development of these collateral pathways results in the dilation of pre-existing capillary veins in the esophagus, stomach, and intestinal tract, leading to the formation of varices [6,7].

Cirrhosis, representing the advanced stage of chronic liver diseases, is an escalating contributor to both morbidity and mortality on a global scale. Alcoholic liver disease, metabolic-associated steatotic liver disease (MASLD), and viral hepatitis represent the three predominant etiological factors leading to liver cirrhosis [8]. Recent data indicate that cirrhosis accounts for an annual global mortality of 12.6 million [9], with 34,200 deaths reported in the United States [10]. It is ranked among the top 14 leading causes of death worldwide. Hepatic cirrhosis is also the predominant etiology of portal hypertension in the Western Hemisphere, whereas schistosomiasis is a prevalent cause in endemic regions, including southern and sub-Saharan Africa, South America, the Caribbean, parts of China, and Southeast Asia [11,12].

The contemporary gold standard for the diagnosis and staging of cirrhotic (sinusoidal) portal hypertension is the hepatic venous pressure gradient (HVPG) measurement. This technique estimates portal pressure by calculating the differential between wedged hepatic venous pressure (WHVP) and free hepatic venous pressure (FHVP) [13]. Under physiological conditions, HVPG values typically range from 1 to <5 mmHg. An HVPG exceeding 5 mmHg denotes the presence of portal hypertension, while an HVPG greater than 10 mmHg is indicative of clinically significant portal hypertension (CSPH), which is associated with the potential development of severe, life-threatening clinical complications [1].

This review aims to provide a comprehensive overview of evolving pharmacological treatments for portal hypertension, alongside an in-depth analysis of its complications and their underlying mechanisms, including current pharmacological management strategies, as well as a study of genetic variations in the development of portal hypertension, polymorphisms and their impact on the response to therapy, and pharmacogenetic considerations in the treatment of portal hypertension.

## 2. Pathophysiology of Portal Hypertension

**Figure 1:**
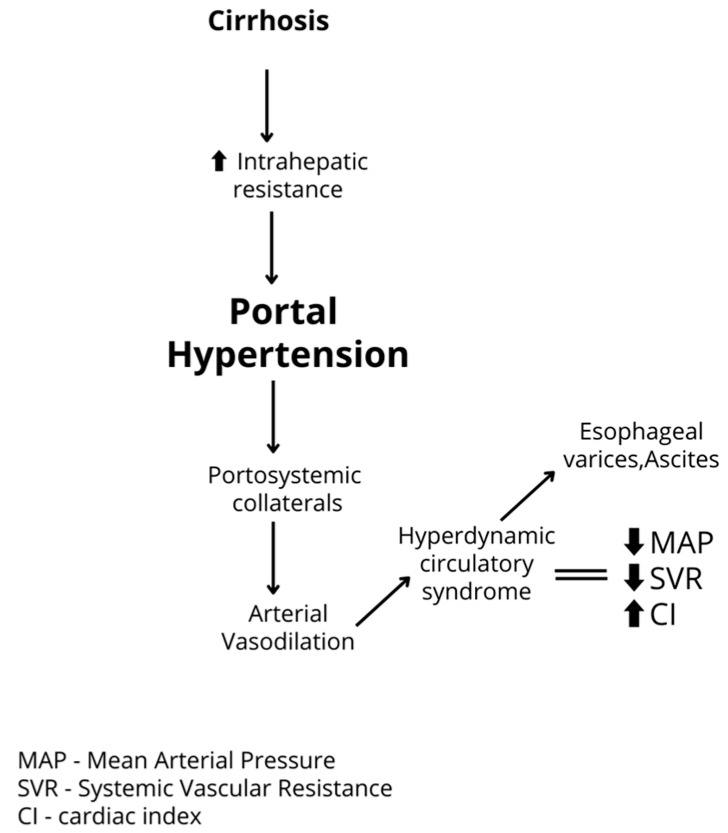
Mechanisms of portal hypertension and hyperdynamic circulatory syndrome in liver cirrhosis.

Cirrhosis is a heterogeneous condition, sub-classified into two primary clinical stages: compensated and decompensated [14,15]. Decompensated cirrhosis is characterized by the onset or presence of clinically apparent complications, such as ascites, hepatic encephalopathy, variceal hemorrhage, or jaundice, [14] and serves as the principal determinant of mortality in cirrhotic patients [15,16]. The development of portal hypertension in cirrhosis involves multiple mechanisms, both intrahepatic and extrahepatic in nature. The primary mechanism is intrahepatic, where increased portal pressure is predominantly caused by elevated resistance to blood flow. This resistance arises from structural alterations in the liver parenchyma, such as fibrosis, disruption of sinusoidal architecture, and microthrombosis within the hepatic vasculature. These pathophysiological changes, including increased intrahepatic vascular resistance and systemic vasodilation, directly contribute to the development of complications such as variceal bleeding, ascites, and hepatic encephalopathy, which are critical factors in the management of portal hypertension (Figure 1).

Additionally, functional or dynamic changes, including endothelial dysfunction and active vasoconstriction, further exacerbate the condition [17]. In compensated cirrhosis with mild portal hypertension, intrahepatic mechanisms are the dominant contributors, making both structural and functional abnormalities promising therapeutic targets at this stage [18].

Fibrogenesis is characterized by the formation of scar tissue comprised of various ECM components [19,20]. Central to the wound-healing response is the activation of myofibroblasts [21,22]. These myofibroblasts originate from hepatic stellate cells (HSCs), which are mesenchymal cells situated in the perisinusoidal space between hepatocytes and the sinusoidal endothelium [23,24]. Additionally, they synthesize and secrete a range of pro-inflammatory and profibrogenic cytokines, as well as various ECM components [25,26]. These structural abnormalities are responsible for approximately 70% of the increased intrahepatic resistance. Effective treatment of the underlying etiology is critical in cirrhotic patients, as regression to earlier stages of liver fibrosis is possible, as demonstrated in cases of hepatitis B following successful therapeutic intervention [27]. The rate of fibrosis progression depends on environmental and genetic factors and the underlying cause of the insult [28,29]. Therapeutic strategies targeting fibrosis have the potential to delay the progression of portal hypertension or even induce regression to a pre-cirrhotic stage [30].

Understanding these pathophysiological mechanisms provides a critical foundation for the development of targeted pharmacological treatments that not only address portal hypertension but also prevent and manage its life-threatening complications.

## 3. Genetic Variations and the Development of Portal Hypertension

The pathophysiology and severity of steatohepatitis, a cause of portal hypertension, appear to result from a complex interplay of genetic factors (such as polymorphisms in PNPLA3, TM6SF2, MBOAT1, or HSD17B13), associated comorbidities (including obesity, type 2 diabetes mellitus, and gut dysbiosis), as well as environmental and behavioral influences (such as socioeconomic status, diet, and physical activity) [31,32,33,34,35,36,37]. Such complexity necessitates a personalized approach to prevention and treatment, addressing not only the underlying genetic and metabolic contributors but also environmental and lifestyle modifications to effectively manage and mitigate disease progression [38].

Therefore, it is crucial to prioritize future research on genetic polymorphisms, focusing not only on those related to non-alcoholic steatohepatitis (NASH) but also on polymorphisms directly involved in the pathogenesis of portal hypertension. In this context, we analyzed genetic variants in various segments that may have the potential to improve the therapeutic management and development of portal hypertension.

### 3.1. Vascular Growth and Angiogenesis Genes

Vascular endothelial growth factor C (VEGF-C), a member of the platelet-derived growth factor family, acts as a ligand for receptor tyrosine kinases VEGF-R3, and VEGF-R2, with a higher binding affinity for VEGF-R3 [39]. The primary physiological role of VEGF-C is the induction of lymphangiogenesis. However, recent studies have indicated that VEGF-R3 also plays a role in angiogenesis [40,41].

Excessive VEGF-C expression has been documented in various cancers, including hepatocellular carcinoma (HCC) [42,43,44]. Notably, HCC patients carrying the VEGF-C polymorphic genotype rs3775194 GC/CC exhibit a lower prevalence of positive liver cirrhosis tests, while individuals with the VEGF-C polymorphic genotype rs7664413 CT/TT are at an elevated risk of developing advanced clinical stages (III or IV) compared to those with the homozygous C/C genotype [45]. Patients with certain genetic variants are at increased risk of developing advanced liver disease, a condition closely linked to higher rates of portal hypertension complications. Early identification of VEGF-C polymorphisms in such cases may allow for targeted therapeutic strategies, such as angiogenesis-targeted therapy. By modulating pathological angiogenesis, the therapeutic approach could reduce intrahepatic vascular resistance, improve liver perfusion, and potentially slow the progression of liver fibrosis [30,46]. Moreover, it may help alleviate complications of portal hypertension, such as variceal bleeding or ascites, which will contribute to improving overall treatment outcomes for patients. However, careful monitoring and further studies are necessary to assess the potential risks associated with the use of these drugs, such as impaired wound healing related to the long-term use of angiogenesis inhibitors [47].

This suggests to us that polymorphisms in the VEGF-C gene, such as rs3775194 and rs7664413, may play a significant role in the progression of liver diseases and the development of portal hypertension. They may be important not only in the context of PH development itself but also in assessing the risk of progression to advanced stages of the disease, which directly impacts therapy selection. However, studies are needed to prove this assumption.

### 3.2. Inflammatory Cytokine Polymorphisms

Cao et al. found that the IL-10 rs1800896 polymorphism was correlated with an increased risk of CL, especially in individuals with chronic hepatitis B [48]. This polymorphism could be significant in the context of PH, as it may exacerbate liver damage, which leads to increased portal pressure. Similarly, Hennig et al. indicated that polymorphisms in the IL-10RA gene may play a modulatory role in the outcome (including the severity of fibrosis and overall inflammation) of hepatitis C virus infection [49]. Additionally, individuals with a genotype with certain variants may have a more severe inflammatory process in the liver, which can accelerate disease progression and worsen treatment outcomes. Knowledge of these genetic variations can help in selecting appropriate anti-inflammatory and immunosuppressive therapies and in assessing the risk of complications such as advanced liver damage. Given the role of chronic liver diseases such as HBV and HCV in the development of portal hypertension, these findings suggest that genetic variations in IL-10 and its receptor may play a role in modulating the progression of liver fibrosis, and by extension, portal hypertension.

Furthermore, a meta-analysis has suggested that polymorphisms in IL-10, particularly at positions −592 and −1082, are associated with an increased risk of chronic liver disease [50]. However, these associations require validation through further large-scale, comprehensive studies to confirm their clinical significance and elucidate their potential role in portal hypertension pathogenesis.

In addition to IL-10, IL-6 has been implicated in the development of liver diseases and subsequent portal hypertension. The concentration of IL-6 in serum was significantly higher in cases of alcoholic or non-alcoholic liver cirrhosis and toxic hepatitis compared to the control group [51]. Genotyping of the IL-6 rs1800796 polymorphism reveals that carriers of the CC genotype exhibit higher levels of IL-6 mRNA than those with the CG/GG genotype [52,53]. These findings support the hypothesis that IL-6 polymorphisms may contribute to susceptibility to liver diseases, including those that lead to PH. This was further corroborated by studies showing an association between the GC genotype of IL-6 rs1800796 and the presence of chronic HBV infection [54]. Additionally, other study findings showed that IL-6 polymorphisms rs1800795 and rs1800796 may be potential genetic factors in the development of liver diseases [55].

Variability in the TNFA promoter region, including the -308G>A (rs1800629) polymorphism, has been associated with altered TNF-alpha production, which may influence the severity of liver damage. In some studies, the TNFA-238G>A (rs361525) polymorphism has been linked to increased TNF-alpha production, though other studies have not observed such an effect [56,57,58,59]. A meta-analysis suggests that the TNFA-238G>A polymorphism may significantly contribute to the risk of alcoholic liver disease (ALD) [60].

To further elucidate the relationship between genetic polymorphisms and the development of portal hypertension, it is essential to combine and analyze existing studies on IL-6, IL-10, and TNF-alpha polymorphisms.

### 3.3. Polymorphisms in Antioxidant and Vascular Regulation Genes

Schwab et al. analyzed two single nucleotide polymorphisms of SOD1 (rs1041740 and rs3844942) in a cohort of 49 patients with liver cirrhosis who underwent liver transplantation [61]. While no association was identified between rs3844942 and complications of liver cirrhosis, a significant correlation was observed between rs1041740 and the occurrence of ascites and spontaneous bacterial peritonitis (SBP) in the discovery cohort of patients with liver cirrhosis [61]. These findings underscore the potential involvement of oxidative stress pathways in the progression of portal hypertension and associated sequelae.

Similarly, Annicchiarico et. al. conducted a study aimed at evaluating the influence of the ACE I/D polymorphism on portal pressure [62]. The findings revealed that the presence of the ACE I allele was significantly associated with higher HVPG values (18.7 ± 6.4 mmHg vs. 10.3 ± 6.3 mmHg; *p* < 0.001) [62]. Additionally, patients carrying the ACE I allele demonstrated a higher frequency of large gastroesophageal varices (59.3% vs. 25.0%; *p* < 0.05) and an increased incidence of variceal bleeding (63.0% vs. 29.2%; *p* < 0.05) [62]. These results underscore the potential role of ACE I/D polymorphism in the progression and complications of portal hypertension.

### 3.4. Polymorphisms in Genes Related to Liver Fibrosis and Lipid Metabolism

Genetic variants associated with liver fibrosis and metabolic regulation further highlight the intricate genetic underpinnings of portal hypertension. The G allele of rs738409, encoding the I148M variant of the patatin-like phospholipase domain-containing protein 3 (PNPLA3), has been independently associated with NASH in the general population [63,64]. Furthermore, the G allele has been linked to increased liver stiffness measurements (LSM) (95% CI: 1.435–3.979; *p* < 0.001) and higher odds of advanced chronic liver disease and CSPH (aOR: 1.685; 95% CI: 1.180–2.406; *p* = 0.004) [65]. This highlights the allele’s potential impact on fibrosis progression and vascular resistance in the liver.

Similarly, the Pi*Z allele of SERPINA1 (rs17580), encoding Serpin Family Member 1, has been associated with liver cirrhosis, inflammatory activity, and advanced fibrosis [66]. The Pi*Z allele was not correlated with increased liver stiffness or the presence of advanced chronic liver disease (ACLD), it was independently associated with higher odds of CSPH (aOR: 2.122; 95% CI: 1.067–4.218; *p* = 0.032) [65]. Moreover, the coexistence of both the PNPLA3 G allele and SERPINA1 Pi*Z variant further amplified the risk of ACLD and CSPH, highlighting a synergistic effect of these genetic variants in the progression of liver disease and portal hypertension [65].

Semmler et al. conducted a study analyzing the impact of farnesoid X receptor single nucleotide polymorphisms (SNPs) on liver decompensation and mortality in patients with cirrhosis and portal hypertension [67]. Two FXR SNPs (rs56163822 and rs35724) were analyzed in a cohort of 402 patients. The results indicate that the rs35724 allele is associated with a reduced risk of ascites and mortality [67]. These findings are significant in the context of future therapeutic strategies, as FXR receptor agonists may soon have potential clinical applications. If validated, these genetic markers could facilitate personalized medicine, allowing clinicians to identify patients more likely to benefit from FXR-based therapies. Moreover, integrating genetic testing for FXR SNPs into routine clinical assessments could help guide treatment decisions and optimize patient care.

## 4. Complications of Portal Hypertension

**Figure 2:**
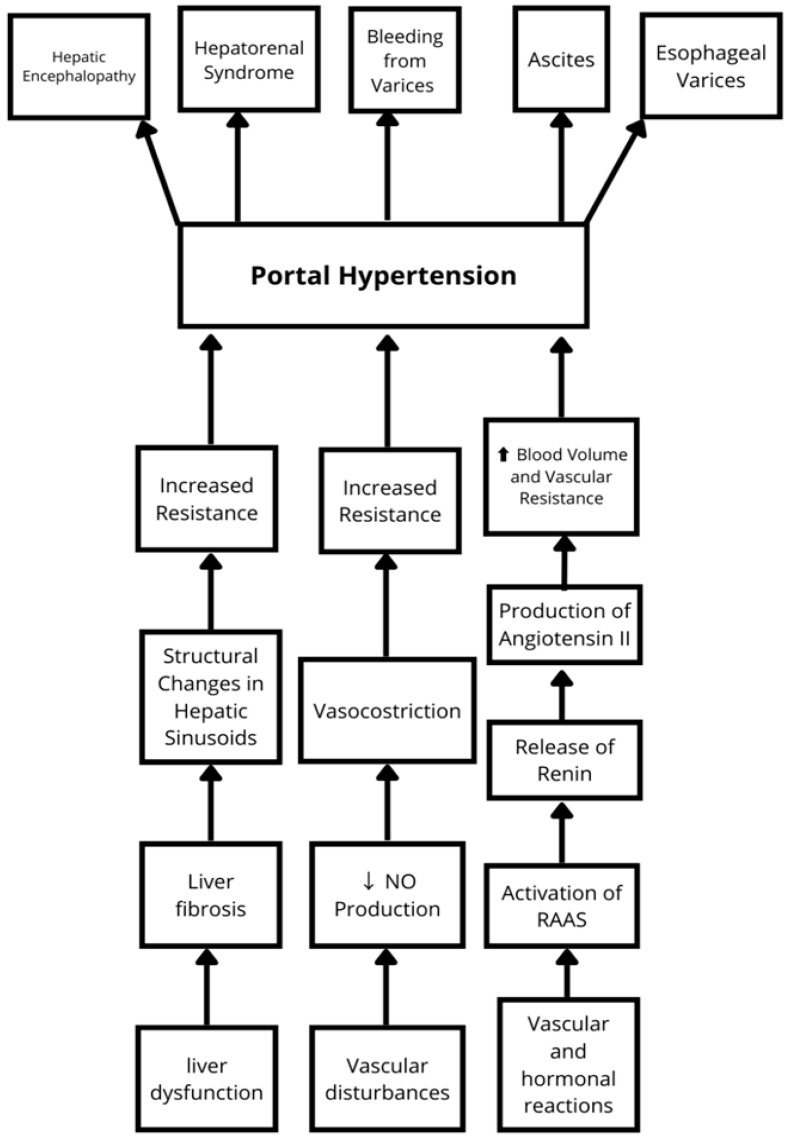
The pathophysiological mechanisms underlying PH and its subsequent clinical consequences.

The pharmacological management of portal hypertension aims to reduce portal pressure to prevent its complications. The pathophysiological pathways responsible for the development of hypertension are summarized in Figure 2. The following paragraphs will elaborate on selected clinical sequelae.

### 4.1. Portopulmonary Hypertension (PoPH)

PoPH is a critical condition linked to advanced liver disease, characterized by the simultaneous presence of pulmonary arterial hypertension (PAH) and PH [68,69,70]. Prospective studies report a prevalence of 2–6% among cirrhotic patients [71,72,73], with portopulmonary hypertension commonly manifesting in individuals with severe and prolonged portal hypertension [70,74]. In patients with PoPH, right ventricular (RV) remodeling occurs as a compensatory mechanism to counteract the elevated resistance in pulmonary circulation [75,76]. The capacity of the RV to adapt to pulmonary hypertension is contingent upon its ability to enhance systolic performance in response to increased afterload [77,78]. Consequently, RV function emerges as a critical determinant of prognosis in this patient population [79,80,81,82].

### 4.2. Varices

Esophageal and gastric varices are observed in approximately two-thirds of individuals diagnosed with cirrhosis [83,84]. The annual incidence rate of new varices in patients with cirrhosis is estimated to be 8–10%. For patients with pre-existing varices, the progression rate from small to large varices is reported to be between 8% and 12% per year [85,86]. While the incidence rate of varices typically exhibits an upward trend, evidence indicates that varices can be reversible, especially in patients who present with only small varices at baseline [87]. An earlier study demonstrated that variceal progression could be reversed in patients with alcoholic liver cirrhosis following abstinence from alcohol and stabilization of liver injury [88,89]. Similarly, in patients with viral hepatitis, a 12-year cohort study revealed that the majority of individuals who achieved sustained suppression of hepatitis B through nucleos(t)ide analogs experienced regression of esophageal varices [90]. Varices are categorized based on their anatomical location and the extent of vascular involvement, primarily classified as esophageal or gastric varices.

#### 4.2.1. Esophageal Varices

The gastroesophageal junction, where the distal esophagus transitions into the proximal stomach, can be anatomically segmented into four distinct regions: truncal, perforating, palisade, and gastric [91,92]. Of these, the palisade zone is particularly susceptible to variceal formation due to its spontaneous portosystemic anastomoses with the azygos and hemiazygos veins [92].

#### 4.2.2. Gastric Varices

Sarin’s classification of gastric varices categorizes them based on their association with esophageal veins. Accordingly, gastric varices are classified as gastroesophageal varices (GOVs) when there is involvement with the esophageal veins, and as isolated gastric varices (IGVs) when such involvement is absent [84].

#### 4.2.3. Variceal Bleeding

The primary determinants in the development of varices include the chronicity of hepatic injury, the elevation of portal pressure, and the extent of portosystemic shunting [93]. Generally, esophageal varices exhibit a higher frequency of bleeding compared to gastric varices. However, when bleeding occurs in gastric varices, it tends to be more severe [84]. Reports indicate that bleeding occurs in approximately 30–50% of patients with varices [85,94], with mortality rates associated with bleeding episodes ranging from 15% to 30% [93,95]. According to Frank’s modification of Laplace’s law, the risk of variceal rupture is directly proportional to the difference between intravariceal pressure (VAp) and luminal pressure (LUp), as well as the radius of the varix (R). Conversely, the risk is inversely proportional to the thickness of the variceal wall (L) [87]. The primary risk factors for the initial bleeding event in variceal hemorrhage include large variceal size with red color signs, a hepatic venous pressure gradient exceeding 16 mmHg, coagulopathy, and the severity of the underlying liver disease as assessed by Child–Pugh or model for end-stage liver disease (MELD) scores [96].

In patients experiencing active bleeding, the foremost priorities are to achieve hemorrhage control, ensure hemodynamic stability, and prevent complications arising from the hemorrhage [87].

### 4.3. Ascites, Fluid Retention

The pathophysiology of ascites is multifaceted, involving at least three principal mechanisms: portal hypertension, splanchnic and peripheral arterial vasodilation, and neurohumoral activation [97,98,99,100]. Cirrhotic ascites primarily arises from impaired renal sodium excretion, which results in a positive sodium balance. This sodium retention subsequently leads to water retention and expansion of the extracellular fluid volume [101]. The reduction in sodium excretion is primarily attributable to arterial vasodilation, which induces neurohumoral responses such as activation of the renin–angiotensin–aldosterone system (RAAS) and sympathetic nervous system (SNS). These responses lead to renal vasoconstriction and sodium retention, thereby contributing to the development of ascites and oedema [101,102,103]. Ascites does not manifest without the presence of moderate to severe portal hypertension, typically characterized by a post-sinusoidal pressure gradient exceeding 12 mmHg [98,104]. Portal hypertension induces increased hydrostatic pressure within the hepatic sinusoids, leading to the transudation of fluid into the peritoneal cavity [98]. Consequently, the volume of ascitic fluid is proportional to the extent of hydrostatic pressure [98]. A critical event in the pathogenesis of sodium retention, ascites formation, and renal dysfunction is the development of arterial vasodilation [105]. Excessive production of vasodilators, driven by increased shear stress within the splanchnic circulation and neurohumoral signals transmitted from the liver to the central nervous system, constitutes a major factor in this process. This enhanced vasodilation leads to reduced effective arterial blood volume, which triggers compensatory mechanisms that exacerbate sodium retention and fluid accumulation [97,106].

### 4.4. Kidney Injury and Hepatorenal Syndrome

Acute kidney injury (AKI) in cirrhosis affects approximately 20% of hospitalized patients [107]. AKI is clinically defined by an increase in serum creatinine levels exceeding 133 µmol/L (1.5 mg/dL), an elevation of over 50%, or an absolute increase in serum creatinine greater than 26.4 µmol/L (0.3 mg/dL) (or a 50% increase within 48 h) [108,109,110]. The differential diagnosis of AKI in cirrhosis poses challenges, as it encompasses pre-renal, intra-renal, and hepatorenal syndrome (HRS) [107,110]. Distinguishing HRS from other forms of AKI involves excluding factors such as dehydration, hypotension or shock, intolerance to diuretic therapy, and bacterial infections and is supported by biochemical tests and renal ultrasonographic findings [111]. HRS represents a form of acute or sub-acute renal failure in cirrhotic patients with advanced liver disease, characterized by functional renal impairment due to vasoconstriction of the renal arteries, with preserved tubular function and near-normal renal histology [110,112]. HRS is clinically characterized by a form of prerenal renal failure that fails to improve with volume expansion. It is essential to regard HRS as a diagnosis of exclusion, requiring the elimination of alternative etiologies of AKI in patients with cirrhosis [109,110].

## 5. The Current Approach to the Pharmacological Treatment of Portal Hypertension

Current knowledge regarding the pathophysiology and complications underlying the development of portal hypertension has led to the identification of therapeutic targets, receptors, and mechanisms that can be modulated through the pharmacological agents currently used to treat portal hypertension. Understanding these pathways has enabled the development of therapies aimed at specifically addressing the multifactorial nature of portal hypertension and its associated complications, offering opportunities for targeted interventions.

### 5.1. Beta-Blockers

The treatment of portal hypertension is largely dependent on its etiology. The management of prehepatic portal hypertension, such as that resulting from portal vein thrombosis, differs from the approach to portal hypertension of hepatic origin, caused by liver cirrhosis. Rational pharmacological therapy seeks to mitigate portal pressure (PP) by targeting the underlying pathophysiological mechanisms of portal hypertension [113]. In patients with compensated cirrhosis, the primary objective of treatment is to prevent progression to decompensation.

In 1981, non-selective β-blockers(NSBBs) were initially introduced for the management of recurrent gastrointestinal bleeding in patients with cirrhosis [114]. Subsequently, they were demonstrated to be similarly effective in the primary prophylaxis of variceal bleeding [115]. Numerous clinical trials have since confirmed the efficacy of NSBBs as first-line pharmacotherapy for variceal bleeding [114,116,117,118,119]. NSBBs reduce HVPG through two principal mechanisms: first, by decreasing cardiac output, which subsequently lowers splanchnic blood flow (via cardiac β1-adrenergic blockade), and second, by increasing splanchnic vascular resistance through unopposed vasoconstrictive α-adrenergic activity, thereby reducing portal blood flow (via splanchnic β2-adrenergic blockade) [120,121]. By these mechanisms, NSBBs lower portal pressure and also prevent complications such as variceal bleeding and ascites, common in advanced portal hypertension. However, their efficacy is limited in patients with decompensated cirrhosis, as they may worsen systemic hypotension and renal impairment [122,123,124]. The American Association for the Study of Liver Diseases (AASLD) guidelines, the British Society of Gastroenterology (BSG) guidelines, and the Baveno VI consensus all recommend the use of NSBBs for both primary and secondary prophylaxis of variceal bleeding [93,125,126].

The effectiveness of NSBBs in secondary prophylaxis is supported by studies showing that propranolol, administered for one year, significantly decreases the incidence of recurrent bleeding in patients with cirrhosis who have previously experienced gastrointestinal bleeding [114]. Despite their demonstrated clinical efficacy, traditional NSBBs, such as propranolol and nadolol, are poorly tolerated in approximately 15% to 20% of patients. Furthermore, up to 60% of patients fail to achieve a therapeutically significant reduction in HVPG with these agents [127,128,129]. The adverse effects associated with NSBBs, including headaches, fatigue, asthma, and dyspnea, contribute to treatment discontinuation in approximately 15% of patients enrolled in clinical trials [130,131].

NSBBs can impair the capacity to maintain adequate systemic arterial blood pressure. This diminished blood pressure control, combined with impaired renal perfusion, may contribute to the development of HRS [122,123]. The administration of NSBBs is contraindicated in approximately 15% of cirrhotic patients due to the presence of comorbid conditions such as asthma, chronic obstructive pulmonary disease (COPD), compromised cardiac function, or hypotension [124].

Among the four available NSBBs, timolol is not available in an oral formulation, leaving propranolol, nadolol, and carvedilol for clinical use. Nadolol and carvedilol offer the advantage of improved patient compliance due to their once- or twice-daily dosing regimen, in contrast to propranolol, which typically requires administration in three divided doses [132]. Nevertheless, extended-release propranolol mitigates this issue and can be administered once daily, similar to nadolol and carvedilol [132]. Carvedilol possesses the additional pharmacological property of α1-adrenergic receptor antagonism within hepatic vessels. This α1-adrenergic blockade results in reduced intrahepatic vascular tone [132], leading to a more pronounced reduction in portal pressure compared to traditional NSBBs [133]. Additionally, the α1-adrenergic blockade of carvedilol contributes to its anti-inflammatory effects by attenuating cytokine-mediated inflammatory responses [134]. It is important to acknowledge that α1-adrenergic receptor antagonism may induce systemic hypotension [87].

Also, carvedilol has been shown to inhibit HSC activation in vivo [135] and possesses antioxidant, antifibrotic, and anti-inflammatory properties, which may offer potential therapeutic benefits for patients with advanced cirrhosis [136]. Although traditional NSBBs have a minimal impact on blood pressure, carvedilol, when administered at standard doses of 25–30 mg/day, has been associated with hypotension and fluid retention [137]. Therefore, doses greater than 12.5 mg/day are not advised for managing portal hypertension, and the use of carvedilol should be cautioned against in patients with ascites unless there is a concurrent need to address systemic hypertension [138,139].

The objective is to achieve a reduction in HVPG to below 12 mmHg or at least 20% below baseline levels. This reduction is associated with a decreased risk of variceal bleeding, rebleeding, ascites formation, SBP, and hepatorenal syndrome, as well as an improvement in overall survival [113].

In compensated cirrhosis, addressing the underlying cause, such as administering direct-acting antivirals for hepatitis C, can delay the onset of decompensation and potentially reduce HVPG, particularly in the early stages of cirrhosis characterized by mild portal hypertension [140].

### 5.2. Somatostatin Analogs

The efficacy of somatostatin analogs was demonstrated in a double-blind, randomized, pragmatic trial conducted by D’Amico et al. [141] on 262 patients with cirrhosis. In this study, subcutaneous octreotide was compared with a placebo for the prevention of early rebleeding in patients undergoing therapy for esophageal varices. Out of 198 patients eligible for treatment (with β-blockers and/or sclerotherapy), 97 received octreotide, while 101 were assigned to the placebo group. During the 15-day treatment period, the rebleeding rate was 15% in the octreotide group, compared to 27% in the placebo group. Notably, this difference emerged within the first five days of treatment, when 8% of octreotide-treated patients experienced rebleeding compared to 19% in the placebo group [142]. Somatostatin analogs reduce splanchnic blood flow and portal venous inflow by inhibiting vasodilatory hormones, making them effective for the acute management of variceal bleeding. However, their long-term role in managing portal hypertension remains limited, primarily due to their parenteral administration [143]. Recently, an oral formulation of octreotide has been approved for the treatment of acromegaly [PMID: 37755395]. It could represent an innovative and convenient option for the chronic administration of drugs in patients with portal hypertension, offering a potentially more patient-friendly alternative to traditional parenteral therapies. Further studies in this area are warranted.

### 5.3. Vasopressin Analogs

Advances in understanding the pathophysiology of PH, particularly the identification of splanchnic vasodilation and hyperdynamic circulation as key factors in CSPH, have resulted in the use of vasoconstrictive agents (e.g., terlipressin and octreotide) for the management of acute variceal hemorrhage [125]. Terlipressin, a synthetic peptide prodrug, effectively mitigates portal hypertension-induced ascites by inducing vasoconstriction in the splanchnic arteries, thereby restoring hemodynamic equilibrium [144,145,146,147,148]. Terlipressin has been extensively utilized in Europe and other regions for over two decades, demonstrating cost efficiency and improved survival outcomes [149,150,151,152]. Nevertheless, its short distribution half-life, ranging from 8 to 50 min [151], necessitates continuous intravenous (IV) infusion or frequent IV dosing, thereby restricting its use to acute care and hospital settings. Adverse effects associated with terlipressin include arterial hypertension, gastrointestinal disturbances such as nausea, diarrhea, and abdominal pain, as well as peripheral ischemia, skin necrosis, and headache [153,154,155,156].

The main current therapeutic options discussed in this section are summarized in Table 1, which provides a comprehensive overview of the most widely used treatments, outlining their mechanisms of action, approval date, clinical indications, and pharmacokinetics. This summary highlights their key characteristics and the rationale behind their use in clinical practice.

### 5.4. Anticoagulants

Portal vein thrombosis (PVT) represents a complex clinical entity and is currently recognized as the most common complication affecting the hepatic vasculature [169]. The management of PVT in patients with portal hypertension aims to restore portal venous flow and prevent further thrombosis, which could exacerbate portal hypertension. It is characterized by the thrombotic obstruction of the intrahepatic and/or extrahepatic segments of the portal venous system [170,171]. PVT can lead to partial or complete obstruction of portal venous blood flow, with clinical presentations ranging from asymptomatic findings detected incidentally on abdominal imaging to severe manifestations associated with the hemodynamic consequences of PH [170,171,172,173]. The initiation of anticoagulation therapy is determined by the stage PVT and the clinical context in which it develops [169]. The therapeutic options currently available for the management of PVT include heparins, vitamin K antagonists (VKAs), and direct oral anticoagulants (DOACs).

Heparins, comprising unfractionated heparin (UFH) and low-molecular-weight heparin (LMWH), are the cornerstone of initial anticoagulation therapy. UFH requires intravenous administration, while LMWH is delivered subcutaneously, both of which carry a risk of bleeding complications, particularly in cirrhotic patients [174]. The common clinical practice is to transition to oral anticoagulants following an initial course of LMWH therapy [169].

VKAs, such as warfarin, acenocoumarol, and phenprocoumon, are well-established oral anticoagulants with a bleeding risk profile comparable to that of heparins. However, their use requires regular monitoring of the international normalized ratio (INR) to maintain therapeutic efficacy [175], which in cirrhotic patients does not reflect the actual pro/anticoagulatory equilibrium. In case of the necessity of reversal of their activity freshly frozen plasma or cryoprecipitate must be used, which leads to fluid overload, increase in portal pressure, and ascites.

DOACs have emerged as a valuable alternative to traditional anticoagulation therapies, offering an immediate anticoagulant effect along with consistent pharmacokinetic and pharmacodynamic profiles [169]. Those drugs do not require routine monitoring, and specific reversal agents are available, contributing to their growing use in clinical practice for the management of PVT in non-cirrhotic patients and those with compensated cirrhosis [174,176,177,178,179,180,181,182,183,184,185].

However, in comparison to traditional anticoagulants, DOACs are associated with higher costs (due to the advent of generic formulations, these are expected to drop significantly) and necessitate strict patient adherence. Additionally, their use requires careful consideration in patients with severe acute or chronic kidney disease, as well as those with compromised liver function [186,187].

In a meta-analysis conducted by Valeriani et al. [188], encompassing a substantial cohort of 1475 cirrhotic patients with splanchnic vein thrombosis, anticoagulation therapy with DOACs was associated with enhanced vein recanalization, decreased thrombosis progression, and improved overall mortality. Notably, this therapeutic benefit was achieved without a concomitant increase in the risk of major bleeding events.

In summary, current pharmacological therapies for portal hypertension primarily target the reduction of portal pressure to prevent complications such as variceal bleeding and ascites. On the other hand, in the case of thrombotic causes of PH, anticoagulants are important tools, especially DOACs, which are becoming more accessible and are backed by the availability of specific “antidotes”. Therapies such as NSBBs in primary and secondary variceal bleeding prevention or somatostatin analogs in acute bleeding settings remain the mainstay of treatment. Nevertheless, agents such as statins and AMP-activated protein kinase (AMPK) activators hold promise in addressing the underlying pathophysiological mechanisms, offering new hope for patients with advanced or treatment-resistant portal hypertension.

## 6. Novel Approach to the Pharmacological Management of PH

Advancements in the understanding of the pathophysiology of PH have facilitated the identification of novel therapeutic targets. These targets hold potential for the development of new pharmacological agents that could either enhance the efficacy of NSBBs or serve as alternatives in patients who exhibit insufficient reduction in portal pressure in response to NSBB therapy. Contemporary therapeutic strategies for portal hypertension can be classified according to their mechanisms of action:

(1) Intrahepatic structural mechanisms, aimed at altering hepatic architecture to diminish vascular resistance;
(2) Intrahepatic functional mechanisms, targeting hepatic hemodynamic regulation to reduce intrahepatic vascular resistance and;
(3) Extrahepatic mechanisms, focused on mitigating splanchnic vasodilation and modulating the development of portosystemic shunts.

### 6.1. Pharmacological Agents Acting Through Intrahepatic Mechanisms by Targeting Structural Modifications Within the Liver

Several pharmacological agents targeting key mechanisms involved in the early stages of hepatic fibrogenesis, particularly the activation and transition of HSCs into proliferative, extracellular matrix-secreting myofibroblasts, are currently under investigation [189,190]. Therapeutic strategies aimed at reducing HSC activation include the use of farnesoid X receptor (FXR) agonists, [191] endothelin-A receptor antagonists, [192,193] the amino acid taurine, [194] and angiotensin II type 1 receptor blockers [195,196,197,198,199,200]. Additionally, the inhibition of hepatocyte apoptosis, a critical process contributing to inflammation and fibrosis, is being explored as a therapeutic target, with the pan-caspase inhibitor emricasan undergoing evaluation for its potential to mitigate liver injury and portal hypertension [201,202].

#### 6.1.1. Farnesoid X Receptor (FXR) Agonists

FXR is a bile acid-responsive transcription factor predominantly expressed in the liver and small intestine [203]. FXR governs bile acid synthesis and modulates the expression of genes integral to hepatic lipid and glucose metabolism, as well as those involved in the regulation of inflammation, fibrosis, and vascular homeostasis [204,205]. FXR agonists have demonstrated efficacy in ameliorating liver disease progression and attenuating fibrosis across various etiologies, as evidenced by experimental animal models of cholestatic, toxic, and NASH-induced fibrosis [206,207,208,209,210,211,212,213,214,215,216,217,218]. FXR agonists enhance bile flow, attenuate inflammatory responses, and improve the regulation of carbohydrate and lipid metabolism [219]. In hepatic endothelial cells, FXR agonists downregulate the expression of endothelin-1 (ET-1) while upregulating dimethylarginine dimethylaminohydrolase (DDAH) [218,220,221]. In murine models, FXR agonists have been shown to downregulate the expression of inducible nitric oxide synthase (iNOS) and cyclooxygenase-2 (COX-2), alongside inhibiting cellular migration [222]. FXR agonists may induce apoptosis in activated HSCs, with this effect being closely associated with the ligands’ capacity to activate the FXR pathway [223,224]. Future studies should examine how genetic variations in FXR pathways influence patient response.

Non-steroidal (e.g., PX-20606, GS-9674) FXR agonists have demonstrated efficacy in reducing portal pressure in models of toxic, cholestatic, and NASH-induced cirrhosis, without adverse effects on systemic hemodynamics [219]. In NASH animal models, GS-9674 was also evaluated in combination with propranolol, which proved to be safe and resulted in an additional reduction of mesenteric hyperperfusion [225]. These findings are promising, and an important next step would be conducting clinical studies combining FXR agonists with NSBBs. This approach could validate the theoretical synergy and potential efficacy of this dual therapy, as the two drug classes target distinct mechanisms involved in portal hypertension. FXR agonists, by modulating bile acid metabolism, reducing inflammation, and attenuating fibrosis, may be particularly effective in patients with liver cirrhosis, where these pathological processes are central to the progression of portal hypertension. In parallel, NSBBs decrease portal vein pressure by reducing splanchnic blood flow through β1/2-adrenergic blockade [120]. The combination of these mechanisms could offer a multifaceted therapeutic strategy, addressing both the underlying pathology and hemodynamic consequences of portal hypertension.

The FXR agonist EDP-305 has been reported to reduce fibrosis, inhibit hepatocyte ballooning, and prevent the progression of steatosis [226]. Therefore, while still in clinical evaluation, FXR agonists might be useful in the mesenchymal/inflammatory cause of PH.

#### 6.1.2. The Amino Acid Taurine

Taurine, known for its diverse physiological effects, has been demonstrated to inhibit the activation of HSCs, potentially contributing to a reduction in portal pressure [227]. This hypothesis was investigated in a single pilot study involving 22 patients, primarily with decompensated cirrhosis and baseline HVPG ≥ 12 mmHg. These patients were randomized to receive either 6 g of taurine daily or a placebo. The taurine group exhibited a statistically significant reduction in HVPG, averaging approximately 12% (~2 mmHg) after four weeks of treatment. Taurine was generally well tolerated, with gastrointestinal discomfort and fatigue being the only notable adverse effects, and no significant alterations in systemic hemodynamic parameters were observed [194]. The clinical significance of this HVPG remains to be elucidated, but the relative safety of the intervention is tempting.

#### 6.1.3. Mas Receptor Agonist (AVE0991)

Angiotensin-(1–7), a metabolite of angiotensin II, exerts opposing effects to Angiotensin II in cirrhosis by attenuating collagen synthesis and promoting vasodilation within the splanchnic circulation [228]. A non-peptide agonist of angiotensin-(1–7) has been shown to reduce portal pressure through the upregulation of vasodilatory mechanisms involving nitric oxide (NO) and the attenuation of the Rho-kinase signaling pathway in cirrhotic rat models [229]. Though intriguing in the mechanism of action, due to the early stage of clinical development results from further studies are necessary to conclude the applicability of this treatment in PH.

#### 6.1.4. Angiotensin II Type 1 Receptor Blockers (ARB)

The renin-angiotensin system plays a multifaceted role in the pathogenesis of PH at various levels [230]. Evidence suggests that angiotensin, via the alternative ACE2 pathway, mediates mesenteric vasodilation in cirrhosis, contributing to hyperdynamic circulation [231]. Additionally, the activation of HSCs through the angiotensin II type 1 receptor has been shown to promote fibrogenesis and elevate intrahepatic vascular resistance, exacerbating cirrhosis-related complications [232]. Unfortunately, the initial two randomized controlled trials (RCTs) revealed that the use of angiotensin II receptor blockers (ARBs) failed to produce a significant reduction in portal pressure in patients with cirrhosis [199,233]. Despite the theoretical rationale behind targeting the renin-angiotensin system, these findings underscore the limited efficacy of ARBs in mitigating the hemodynamic alterations characteristic of cirrhotic portal hypertension. A subsequent RCT indicated that an angiotensin II receptor blocker reduced hepatic vascular resistance in patients with compensated cirrhosis, thereby improving portal venous outflow and highlighting its potential therapeutic role in modulating the renin-angiotensin system in this patient population [234]. The addition of candesartan to propranolol demonstrated no significant advantage over propranolol monotherapy in reducing portal pressure [235]. However, the drugs might be considered as additional drugs in patients with arterial hypertension with PH, but are not recommended as a sole therapy in PH.

#### 6.1.5. Endothelin-A Receptor Antagonists

Endothelins are implicated in the pathophysiology of endothelial dysfunction and are also believed to facilitate the activation of hepatic stellate cells [190]. Ambrisentan, a selective endothelin receptor antagonist targeting ET-A receptors, demonstrated a dose-dependent reduction in HVPG in a pilot study involving subjects with cirrhosis, without adversely affecting systemic blood pressure [236]. In contrast, BQ-123, another selective ET-A antagonist, and BQ-788, a selective ET-B antagonist, did not produce any significant changes in HVPG. While it offers a significant drop in HVPG, it is marketed only for pulmonary hypertension.

#### 6.1.6. Caspase Inhibitors

The pan-caspase inhibitor emricasan (IDN-6556) has demonstrated a reduction in hepatocyte apoptosis, inflammation, and liver fibrosis in animal models of liver injury [201,237]. In a proof-of-concept open-label trial involving 24 patients with cirrhosis and portal hypertension, post-hoc analysis revealed that a four-week treatment with emricasan led to a significant reduction in HVPG, but only in patients with a baseline HVPG ≥ 12 mmHg [202]. A clinical trial (NCT02960204) is investigating the effects of three different doses of emricasan (5 mg, 25 mg, and 50 mg twice daily) on HVPG in patients with NASH cirrhosis and HVPG > 12 mmHg. Furthermore, in other phase-2 studies, emricasan showed improvements in INR and total bilirubin levels in a subgroup of cirrhotic patients with MELD scores ≥ 15 [238]. The results of these studies are anticipated to enable clinical conclusions.

### 6.2. Pharmacological Agents That Exert Their Effects via Intrahepatic Mechanisms by Modulating Hepatic Functional Blood Flow Dynamics

Functional resistance to blood flow, attributable to elevated vascular tone, constitutes approximately 30% of the total vascular resistance [239]. These functional alterations have been linked to sinusoidal endothelial dysfunction, [17] characterized by a reduced synthesis of NO. This impairment is attributed to decreased expression or activation of eNOS [240,241]. This category encompasses substances that could potentially be employed in the future for the management of portal hypertension. The most promising candidates include those discussed below.

#### 6.2.1. Statins

Statins, widely studied and potentially the most promising therapy for PH, function as inhibitors of 3-hydroxy-3-methylglutaryl coenzyme A reductase (HMG-CoA-R), commonly used to treat hyperlipidemia [230]. However, their therapeutic potential extends beyond cholesterol-lowering, with pleiotropic effects including reduced vascular inflammation and oxidative stress, decreased thrombosis, and enhanced NO production in endothelial cells via inhibition of small G-proteins Rho and Rac [242,243]. These mechanisms position statins as a key candidate for future PH management. Statins enhance NO production by promoting the synthesis and phosphorylation of eNOS in the liver, effectively targeting and ameliorating endothelial dysfunction [240,244,245]. A randomized double-blind study (NCT01282385) is investigating the combination of simvastatin and carvedilol in patients with cirrhosis and CSPH, though its results remain unpublished. While results are pending, another randomized trial demonstrated that adding simvastatin significantly reduced HVPG by −16% compared to −11% in the placebo group (*p* < 0.05) [244]. Future research should focus on evaluating the synergistic effects of statins combined with other therapies, such as β-blockers or AMPK activators, to enhance portal pressure reduction. Furthermore, combining them with PDE5 inhibitors, known to potentiate nitric oxide-mediated vasodilation, could provide dual benefits by targeting both vascular tone and endothelial dysfunction. These trials aim to demonstrate acute reductions in portal pressure. While preclinical data have suggested their potential efficacy, ongoing studies have yet to provide definitive results, making their clinical impact promising but as yet unverified (NCT02344823). Similarly, metformin, widely recognized for its metabolic effects, is currently being evaluated in a clinical trial (NCT06687265) for its potential role in managing portal hypertension. While early findings from preclinical studies and phase I trials are encouraging, robust clinical data confirming its efficacy and safety in this context are still lacking. Despite the absence of published results, these approaches represent areas of significant ongoing interest and hold considerable promise for future therapeutic strategies. Early-phase clinical trials have suggested that the addition of statins to conventional treatments may provide further benefits, particularly in patients who are non-responders to conventional therapies. The early administration of statins has demonstrated the ability to mitigate liver fibrosis in animal models during the initial stages of cirrhosis by reducing HSC activation; this antifibrotic effect has been observed with various statins, including atorvastatin [246] and simvastatin [247]. Additionally, statins have been demonstrated to suppress neoangiogenesis and collateral blood flow in experimental models of cirrhosis by attenuating this same non-canonical hedgehog pathway [248].

An initial exploratory study involving a limited cohort of patients with cirrhosis and baseline hepatic venous pressure gradient ≥ 12 mmHg demonstrated that a single 40 mg dose of simvastatin significantly reduced hepatic resistance by 15%, facilitating an increase in hepatic blood flow, without inducing notable changes in HVPG or systemic hemodynamics [249]. A systematic review and meta-analysis encompassing 13 studies on the use of statins in patients with chronic liver disease revealed significant clinical benefits [250]. Statin therapy was associated with a 46% reduction in the progression of chronic liver disease to decompensated cirrhosis, a 46% decrease in overall patient mortality, and a 26% reduction in both the risk of variceal bleeding and the progression of portal hypertension in cirrhotic patients [250]. A meta-analysis of three randomized controlled trials evaluated the impact of statins on cirrhosis-related complications, including variceal bleeding [251]. The analysis revealed a hazard ratio (HR) of 0.73 (95% CI 0.59–0.91) for reducing the risk of variceal bleeding in patients treated with statins. These findings underscore the potential of statins not only in lowering portal pressure but also in mitigating life-threatening complications associated with portal hypertension [251]. In a recent clinical trial, the addition of simvastatin to NSBB therapy demonstrated a reduction in overall mortality among patients, although it did not significantly lower the rate of variceal rebleeding [252].

Existing data suggest that certain patients with cirrhosis may be too ill to benefit from statin therapy and may be at increased risk of statin-related complications. A recent study found that statins provide survival benefits; however, among patients with Child–Pugh class B, a relatively high incidence of rhabdomyolysis was observed (three patients in the group receiving 40 mg of simvastatin daily developed rhabdomyolysis) [252]. Choosing a water-soluble statin (e.g., rosuvastatin) instead of lipid-soluble agents that have undergone less liver metabolism might be a solution. Patients with advanced cirrhosis may experience more harm than benefit from initiating or continuing statin therapy. Therefore, the majority of survival benefits appear to be concentrated in patients with Child–Pugh class A, with clinically significant portal hypertension or without, but without other signs of liver failure [253]. Contraindications to statin use include prior statin sensitivity or documented statin-induced hepatotoxicity. Furthermore, data suggest that statins should likely not be prescribed to patients with Child–Pugh classes B and C due to the increased risk of statin-induced complications [253]. However, studies assessing the optimal dosing and duration of statin therapy, as well as its role in combination with other treatments, are urgently needed to solidify their place in the treatment of portal hypertension.

#### 6.2.2. Inhibitors of PCSK9 (Proprotein Convertase Subtilisin/Kexin Type 9)

In recent years, inhibitors of PCSK9 have gained significant attention for their role in managing cholesterol levels, especially in patients with hypercholesterolemia [254,255,256,257]. PCSK9 regulates LDL receptor availability, which is crucial for controlling cholesterol metabolism [258,259,260]. By lowering LDL cholesterol levels and modulating vascular dynamics, PCSK9 inhibitors alleviate hemodynamic disturbances in portal hypertension, potentially enhancing liver circulation and reducing intrahepatic vascular resistance [261].

However, the existing literature supports the idea that these inhibitors, by modulating endothelial dysfunction, may have a synergistic effect when used in combination with other therapies, such as statins [262]. This combination could enhance outcomes by targeting both the lipid profile and endothelial dysfunction, commonly observed in liver disease and portal hypertension [263]. Additional research is needed to confirm the clinical benefits of this approach. The combination of PCSK9 inhibitors with statins may improve liver hemodynamics by targeting both cholesterol metabolism and vascular resistance, making it a promising area for future research. Another promising approach is the combination of PCSK9 inhibitors with traditional therapies such as NSBBs. This combination is advantageous due to its complementary mechanisms of action. NSBBs reduce splanchnic blood flow by increasing splanchnic vascular resistance through unopposed vasoconstrictive α-adrenergic activity, thereby decreasing portal blood flow [120,121]. Meanwhile, PCSK9 inhibitors regulate cholesterol metabolism by enhancing LDL receptor availability, resulting in lower LDL cholesterol levels and potentially improving vascular dynamics [261]. These effects may synergistically reduce portal pressure, providing a novel strategy for managing portal hypertension.

Although PCSK9 inhibitors have not yet been extensively studied in the context of portal hypertension, their effects on improving lipid metabolism may suggest their potential benefits in treating this condition.

Thus, while preliminary data on PCSK9 inhibitors are promising, rigorous clinical trials are essential to determine their role in the management of portal hypertension and their potential as adjunctive therapies in the treatment of liver diseases. By focusing on randomized controlled trials and incorporating PCSK9 inhibitors into the therapeutic arsenal for portal hypertension, we can move closer to an evidence-based approach for improving outcomes in these challenging patients. Additionally, one should remember that the relatively high cost of the therapy might limit its usage in real-life scenarios.

#### 6.2.3. PDE5 Inhibitors

In the context of cirrhosis, the synthesis of NO by endothelial cells is diminished, leading to a reduction in cyclic guanosine monophosphate (cGMP) production, which is subsequently degraded to 5′-guanosine monophosphate (5′-GMP) by hyperactive phosphodiesterase-5 (PDE-5) [264,265]. PDE-5 inhibitors act to inhibit the conversion of cGMP to its inactive form, thereby prolonging the vasodilatory effects mediated by the NO-cGMP signaling pathway [266]. Experimental studies utilizing rodent models of biliary cirrhosis have demonstrated that PDE-5 inhibitors enhance sinusoidal blood flow and decrease hepatic vascular resistance by improving NO bioavailability [267]. Furthermore, a phase II clinical trial indicated that the PDE-5 inhibitor udenafil achieved approximately a 20% reduction in portal pressure in acute scenarios without adversely affecting systemic blood pressure [268]. However, contrasting findings have emerged from other investigations, which reported no beneficial impact of PDE-5 inhibitors on portal hypertension and indicated a deterioration in systemic hemodynamic parameters, including renal function [269]. Nevertheless, a potential therapeutic group of patients might include those with complicating pulmonary hypertension; therefore, further focused study in such a population is warranted.

#### 6.2.4. Thalidomide

Thalidomide, an immunomodulatory agent that inhibits the tumor necrosis factor-alpha pathway, thereby attenuating inflammation, [270,271] was investigated in a small cohort of 12 patients with alcohol-related cirrhosis and CSPH. Following two weeks of treatment, the study observed a notable reduction in HVPG, with portal pressure decreasing from an average of 19.7 mmHg to 12.2 mmHg; additionally, five out of six patients exhibited a reduction in HVPG exceeding 20% [272]. Somnolence was the primary adverse event reported during the trial, with no significant alterations in systemic arterial pressure noted [230]. The available data are far from enough to conclude the use of thalidomide in PH, but the toxicity poses a great threat to such therapy.

#### 6.2.5. Anti-Fibrotic Drugs

Activated HSCs represent the primary cellular source of myofibroblasts within the liver, responsible for the overproduction of extracellular matrix components, a hallmark of liver fibrosis [273]. Transforming growth factor β1 (TGF-β1) plays a pivotal role in the activation of HSCs, driving fibrosis through various mechanisms [274]. Numerous therapeutic strategies have been explored to counteract this pathway. Monoclonal antibodies, such as fresolimumab, which is currently undergoing phase II clinical trials, have been developed to neutralize all isoforms of TGF-β [275]. Additionally, lysyl oxidase-like 2 (LOXL2), a key enzyme involved in collagen cross-linking, makes collagen fibers more resistant to degradation. Simtuzumab, a monoclonal antibody targeting LOXL2, [276] initially showed promise in preclinical studies but ultimately failed to demonstrate efficacy in phase II randomized controlled trials for primary sclerosing cholangitis, NASH-related fibrosis, cirrhosis, and HCV/HIV co-infections [276,277,278].

Peroxisome proliferator-activated receptors (PPARs), specifically PPAR-α, β, and γ/δ, are nuclear hormone and fatty acids receptors that play a crucial role in regulating cholesterol synthesis. Dysregulation of PPARs is implicated in processes such as inflammation, insulin resistance, and fibrogenesis [274]. Reactivation of these receptors has demonstrated promising outcomes in reversing liver fibrosis, reducing inflammation, ameliorating steatosis, and addressing extrahepatic complications associated with chronic liver diseases, highlighting their therapeutic potential in targeting multiple pathogenic pathways [279,280,281,282,283,284,285]. Among the various drugs targeting different PPAR isoforms, the pan-PPAR agonist lanifibranor, an indole sulfonamide derivative, has garnered considerable attention due to studies demonstrating its significant effects in experimental models of cirrhosis, highlighting its potential therapeutic value [286]. Additionally, clinical studies indicate that lanifibranor may facilitate fibrosis regression in patients with NASH; however, further research is required to validate its therapeutic efficacy in individuals with advanced-stage fibrosis [194]. PPARγ has the potential to inhibit splanchnic angiogenesis and the development of portosystemic collaterals, as evidenced by preclinical studies utilizing aleglitazar, a dual PPAR α/γ agonist [279]. PPARs modifying drugs seem promising in the course of advanced phases of MASLD and, therefore, may be used in the prevention of PH.

#### 6.2.6. Recombinant Human Manganese Superoxide Dismutase (rMnSOD)

Reduced activity of superoxide dismutase (SOD) has been observed in cases of cirrhosis [287]. Administration of rMnSOD to cirrhotic rats has been shown to lower portal pressure by decreasing hepatic vascular resistance, while maintaining splanchnic blood flow, indicating its potential as a novel antioxidant therapy for portal hypertension [288]. No human studies have been performed so far, and therefore it is hard to make a conclusion, but the use of recombinant proteins, due to their cost in a common disease, is doubtful.

#### 6.2.7. Specific NOX Inhibitor (GKT137831)

NADPH oxidase (NOX) represents a key enzymatic source of reactive oxygen species (ROS) and plays a critical role in determining the redox balance within vascular tissues [289]. The NOX enzyme family consists of seven isoforms, including NOX1 to NOX5 and DUOX1/2. Of these, NOX1, NOX2, NOX3, and NOX5 predominantly generate superoxide anions (O2−), while DUOX1 and DUOX2 are primarily involved in the production of hydrogen peroxide (H2O2) [290]. This differential ROS production is significant in the context of vascular oxidative stress and endothelial dysfunction. Research indicates that oxidative stress, characterized by elevated ROS generation, plays a pivotal role in the pathogenesis of key complications associated with PHT, such as hyperdynamic circulatory syndrome and the development of portal-systemic collaterals [288,291]. NADPH oxidase is central to this process, and while the inhibitor apocynin has been shown to reduce ROS production, its clinical utility is limited by significant off-target effects due to its lack of specificity [290]. More recently, the development of a selective NOX inhibitor, GKT137831, has shown promise [292]. GKT137831 effectively reduces ROS production without impairing NOX2-mediated phagocyte function, suggesting it offers a superior therapeutic profile compared to broader NOX/ROS inhibitors [290]. In a study conducted on rats, [290] inhibition of NOX1/4 using GKT137831 significantly reduced portal pressure, improved hyperdynamic circulation, mesenteric angiogenesis, and arterial hyporesponsiveness in rats with portal hypertension. These findings suggest that pharmacological inhibition of NOX1/4 activity could represent a promising future therapeutic strategy for the treatment of portal hypertension and its associated complication.

#### 6.2.8. Anti-Angiogenics

Impaired microcirculation, driven by distorted intrahepatic circulation, sinusoidal pseudo-capillarization, and fibrosis, stimulates the expression of hypoxia-inducible factors (HIFs) and inflammation, resulting in the release of angiogenic factors such as VEGF, Placental growth factor (PlGF), and PDGF [274]. Elevated portal pressure induces stretching of liver sinusoidal endothelial cells (LSECs), leading to phenotypic dysregulation and activation of the Raf/MEK/ERK signaling pathway [293,294]. Among various anti-angiogenic agents, compounds such as sorafenib [295], sunitinib [296], regorafenib [297], and bivanib [298] have been shown to effectively reduce portal pressure, mitigate splanchnic neovascularization, and decrease portosystemic shunting [274]. Sorafenib, the first multikinase inhibitor demonstrated to enhance overall survival in HCC patients [299], exerts anti-angiogenic effects by inhibiting the autophosphorylation of multiple receptor tyrosine kinases, including VEGFR1, 2, and 3; PDGFRβ; c-Kit; and RET, while also inhibiting various Raf kinase isoforms [300]. In cirrhotic rat models, sorafenib administration led to a 25% reduction in portal pressure and an 80% decrease in splanchnic neovascularization [46].

Glycyrrhizin represents another compound with anti-angiogenic properties that holds potential for future application in the treatment of portal hypertension. Glycyrrhizin, the principal bioactive constituent derived from licorice root extraction, has been extensively utilized in traditional herbal medicine across Asia for its anti-tumor, anti-inflammatory, and anti-viral properties [301]. Furthermore, a clinical trial has indicated that exposure to licorice is associated with increased large arterial stiffness and systemic vascular resistance [302]. Glycyrrhizin and its metabolite, glycyrrhetinic acid, have been shown to mitigate bile-induced hepatotoxicity in rodent models by inhibiting both apoptosis and necrosis of hepatocytes [303]. In studies involving hepatic injury in rats, glycyrrhizin administration resulted in a significant reduction in plasma levels of aspartate aminotransferase (AST) and alanine aminotransferase (ALT), which serve as biomarkers of liver damage, in comparison to the vehicle control group [304,305]. Moreover, research has demonstrated that glycyrrhizin effectively suppresses angiogenesis. Specifically, glycyrrhizin inhibited tumor growth and angiogenesis in vivo, while also attenuating the migration, invasion, and tube formation of endothelial cells [306]. Additionally, another study indicated that glycyrrhizin inhibited the angiogenic activity of endothelial cells and further suppressed both tumor growth and neovascularization in murine models [307].

#### 6.2.9. Rho-Kinase Inhibitors

Rho-kinase (ROCK) activity is significantly linked to the activation of HSCs and the development of endothelial dysfunction [308]. A clinical investigation into fasudil, a specific ROCK inhibitor, demonstrated a rapid decrease in HVPG [309]. However, this treatment was also associated with systemic side effects, including a marked reduction in arterial pressure and systemic vascular resistance [309]. Future approaches may involve targeted delivery systems specifically designed to address HSCs, thereby minimizing these adverse effects [310].

#### 6.2.10. Tetrahydrobiopterin (BH4)

A deficiency in tetrahydrobiopterin (BH4) leads to the uncoupling of eNOS, resulting in reduced NO production, which is a critical factor in the development of endothelial dysfunction [311]. An experimental investigation demonstrated that treatment with BH4 significantly enhanced eNOS activity and substantially decreased portal hypertension in cirrhotic rats [311].

#### 6.2.11. JAK2 Inhibitor (Ruxolitinib)

Angiotensin II exerts its effects through the angiotensin II-type-I receptor, activating the RHOA/Rho-kinase signaling pathway via Janus kinase 2 (JAK2) in extrahepatic vasculature [312]. In experimental models, the administration of a JAK2 inhibitor has been shown to decrease hepatic vascular resistance, thereby alleviating portal hypertension [273]. Ruxolitinib, a dual inhibitor of Janus kinases 1 and 2, was administered to a patient with cirrhosis who required this treatment following bone marrow transplantation for steroid-refractory graft-versus-host disease. In an acute setting, ruxolitinib effectively reduced the HVPG from 11 mmHg to 6 mmHg [313].

#### 6.2.12. AICAR

AICAR (5-aminoimidazole-4-carboxyamide ribonucleoside) is an agonist of the AMPK pathway. Previous studies showed that AICAR treatment reduced tubulointerstitial and interstitial fibrosis [291,314]. A conducted study demonstrated that AICAR, an agonist of the AMP-activated protein kinase pathway, effectively reduces portal venous pressure (PVP) in rats with established portal hypertension and liver cirrhosis [315]. The findings indicated that AICAR not only attenuated liver cirrhosis in the bile duct ligation (BDL) rat model but also improved intrahepatic vascular tone without inducing adverse effects on systemic circulation [315]. In acute AICAR administration experiments, hepatic sinusoid dilation and a reduction in intrahepatic resistance were observed, as evidenced by intravital fluorescence microscopy [315]. It has also been demonstrated that AICAR treatment inhibits HSC contractility by activating the AMPK/NO pathway in vitro [315].

#### 6.2.13. Metformin

Animal studies have demonstrated the efficacy of AMPK activators, such as metformin, in reducing HVPG [315,316,317]. Additionally, metformin has been shown to provide beneficial effects in rodents by reducing liver inflammation and improving hepatic fibrosis [316]. In a randomized clinical trial [318], a significant reduction in HVPG was observed in the metformin group (mean: −16%, 95% CI: −28% to −4%), whereas no significant change was noted in the placebo group (mean: 4%, 95% CI: −6% to 14%). Metformin also reduced the WHVP by an average of −15% (95% CI: −25% to −5%), while the placebo group showed no significant change (mean: 2%, 95% CI: −5% to 9%) [318]. This randomized trial demonstrates that a single dose of metformin rapidly decreases HVPG by 16% in patients with cirrhosis and portal hypertension, with a clinically significant reduction in HVPG observed in 46% of patients treated with metformin [318]. While it is tempting to use the drug in PH, one must remember the side effects of metformin and its ability to induce deadly lactic acidosis, which is more common in liver failure. Therefore, vigilant supervision of lactate seems mandatory.

### 6.3. Pharmacological Agents That Operate Through Extrahepatic Mechanisms Function by Inducing Splanchnic Vasodilation and Facilitating the Formation of Portosystemic Collateral Circulation

The extrahepatic mechanisms are activated in response to early-stage PH. These mechanisms involve splanchnic vasodilation, driven by enhanced nitric oxide synthesis within the splanchnic vasculature, as well as the induction of neoangiogenesis [319,320]. Splanchnic and systemic vasodilation result in relative hypovolemia, triggering neurohumoral activation that promotes sodium and water retention. This process induces hypervolemia, elevates cardiac output, and culminates in a hyperdynamic circulatory state, which further augments portal blood flow and contributes to the progression of CSPH [17,321]. This category comprises pharmacological agents that hold potential for future application in the treatment of portal hypertension. The most promising include those discussed below.

#### 6.3.1. Selective Intestinal Decontamination

Recent studies have indicated that patients with cirrhosis experience progressive alterations in gut microbiota [322,323]. This bacterial overgrowth is associated with elevated endotoxin levels and circulating pro-inflammatory cytokines, which may drive the intrahepatic release of endothelin and COX via cytokine stimulation [324]. Given the role of bacterial translocation in sustaining inflammation in cirrhosis, poorly absorbed antibiotics have been investigated as a therapeutic approach for PH, aiming to reduce bacterial overgrowth and translocation, thus mitigating the associated inflammatory cascade [230]. Rifaximin, a minimally absorbed antibiotic, exhibits broad-spectrum antimicrobial activity in vitro, making it a valuable agent for targeting gut microbiota without significant systemic absorption [273]. A small clinical trial involving 13 patients with alcohol-related cirrhosis and decompensation due to ascites demonstrated that rifaximin treatment led to a reduction in cardiac output, increased vascular resistance, and an improvement in eGFR, suggesting a potential amelioration of the hyperdynamic circulatory state, although HVPG was not measured [325].

A study assessing norfloxacin did not demonstrate significant improvement in HVPG but did show an increase in MAP, enhanced vascular resistance, and a reduction in endotoxin levels [326]. Additionally, a post-hoc analysis from a recent RCT involving 291 patients with Child–Pugh class C cirrhosis revealed that long-term use of norfloxacin was linked to improved survival, particularly in patients with low ascites protein levels (<15 g/L) [327]. Due to its simplicity and clinical benefits, in decompensated liver failure GI tract decontamination might serve as an important part of PH therapy.

#### 6.3.2. Splanchnic Vasculature Constricting Agents OCE-205

Terlipressin, a vasopressin analog, received approval from the U.S. Food and Drug Administration in September 2022 for the treatment of adults diagnosed with HRS accompanied by a rapid decline in renal function [328]. This approval marks a significant advancement in addressing the complex pathophysiology of HRS, as terlipressin has demonstrated efficacy in improving renal perfusion by selectively constricting splanchnic vasculature, thereby reducing portal pressure and mitigating kidney injury in the setting of cirrhosis-related renal dysfunction [329]. Terlipressin is associated with notable adverse effects, including tissue hypoperfusion and ischemia, attributable to its potent vasoconstrictive properties, which are mediated by lysine vasopressin’s (LVP) full agonistic activity at the V1a receptor [330,331,332]. Additionally, through stimulation of the V2 receptor, terlipressin can induce water retention, leading to fluid overload and consequent respiratory failure [333,334,335,336,337]. These hemodynamic alterations highlight the need for careful monitoring in patients undergoing terlipressin therapy, especially those with compromised circulatory and respiratory function.

OCE-205 represents a more refined therapeutic strategy by selectively targeting V1a receptors with submaximal activity while avoiding engagement with V2 receptors. This design was inspired by a 1996 hypothesis that posited a single molecule with both agonist and antagonist properties could function as a partial agonist [338]. The selective modulation of the V1a receptor allows OCE-205 to exert vasoconstrictive effects while minimizing the adverse outcomes associated with V2 receptor activation, such as fluid retention and the risk of respiratory failure [329]. OCE-205 is an innovative peptide engineered to modulate the vasopressin receptor system, functioning as a mixed agonist/antagonist at the V1a receptor. This dual activity results in effective partial agonism, providing targeted vasoconstriction with a reduced risk of adverse effects commonly associated with full agonists, thus offering a potentially safer and more efficient therapeutic option [329]. Unlike other arginine vasopressin (AVP) analogs, OCE-205 is designed to avoid V2 receptor activation, thereby preventing water retention. By selectively inducing the desired vasoconstrictive effect, it offers therapeutic potential for managing complications associated with cirrhotic portal hypertension, such as hepatorenal syndrome with acute kidney injury (HRS-AKI) [329]. This targeted approach allows OCE-205 to modulate the underlying pathophysiology of the disease while minimizing undesirable side effects [329].

We can also identify substances that exert their effects through multiple mechanisms of action and cannot be rigidly classified into a single category. These include the following:

#### 6.3.3. Eculizumab

Eculizumab is a monoclonal antibody that specifically targets complement protein C5, blocking its cleavage into C5a and C5b, thereby inhibiting the formation of the terminal complement complex C5b-9 [339]. This action prevents the downstream effects of complement activation, which are critical in immune-mediated pathological processes [340]. Eculizumab has garnered attention as a potential therapeutic option for portal hypertension, particularly due to a reported rare case of idiopathic non-cirrhotic portal hypertension (INCPH) complicating paroxysmal nocturnal hemoglobinuria (PNH) in a 63-year-old patient. In this instance, the patient was effectively treated with eculizumab for non-cirrhotic portal hypertension, highlighting its potential utility in managing similar cases [339].

### 6.4. Other Therapeutic Horizons in PH

We conducted a review of currently ongoing clinical trials using clinicaltrials.gov, focusing on studies with a clear status, excluding those with unknown statuses. The data in Table 2 reveal a wide range of molecules that could potentially be applied in the treatment of portal hypertension. The results of the studies and the effectiveness of the therapies show significant variability. Many of the therapies studied, such as avenciguat, ambrisentan, NCX-1000, and cobiprostone, have not yielded positive results. However, some showed very promising results. BI 685509 was administered to 64 patients with mild or moderate hepatic impairment (Child–Pugh class A) and healthy volunteers [341]. The aim of the study was to assess the safety, tolerability, and pharmacokinetics of this molecule. BI 685509 resulted in adverse events in 5.6% of patients with Child–Pugh class A cirrhosis and 26.3% of patients with Child–Pugh class B cirrhosis, with no serious adverse events observed. Additionally, the compound demonstrated rapid absorption [341].

GR-MD-02 (belapectin), an inhibitor of galectin-3 that reduces liver fibrosis, was evaluated for its safety and efficacy in patients with MASH, liver cirrhosis, and PH [342]. Patients were randomly assigned, in a double-blind manner, to receive biweekly infusions of belapectin at doses of 2 mg/kg (n = 54), 8 mg/kg (n = 54), or placebo (n = 54) for 52 weeks. Unfortunately, no significant difference in HVPG was observed between the 2 mg/kg belapectin group and the placebo group, nor between the 8 mg/kg belapectin group and the placebo group [342]. Another significant finding was the study conducted by Zhu et al., which aimed to elucidate the role and mechanism of the 5-HT 1A receptor (HTR1A) in the portal vein in PH [343]. It was demonstrated that the expression of HTR1A was significantly increased in the hypertensive portal vein of rats with a PH model and in patients with liver cirrhosis [343]. Moreover, alverine was confirmed to be an HTR1A antagonist, and its ability to reduce PP was demonstrated in rats with PH induced by thioacetamide [343]. As a result, HTR1A represents a promising therapeutic target for PH. Progress in this field requires both a deeper understanding of the pathophysiological mechanisms of the disease and an increased number of high-quality and reliable studies that will not only seek effective drugs but, most importantly, ensure safety for patients.

### 6.5. Potential Benefits and Risks of PH Pharmacotherapy

To enhance the understanding of the efficacy and limitations of various therapies for portal hypertension, a comparative analysis of traditional and emerging approaches based on clinical and experimental data has been performed and the results were gathered, as shown in Table 3. Not all studied molecules described previously in this paper are included, due to limited data availability. Molecules for which information was insufficient to allow an objective and comprehensive analysis were omitted from the table.

We believe that the key gaps in research on portal hypertension include the lack of multicenter clinical trials involving combination therapies, where different compounds target distinct molecular pathways and exhibit varied mechanisms of action, such as the integration of NOX inhibitors and FXR agonists with conventional therapies like beta-blockers. Furthermore, such studies should involve large patient cohorts to ensure sufficient statistical power, and their findings must undergo validation in independent populations.

Additionally, identifying novel gene-drug interactions in the treatment of portal hypertension remains a significant challenge. Given the polygenic nature of hypertension, future studies should explore not only individual genetic polymorphisms but also their combined effects and potential interactions. Developing a comprehensive genetic risk score for patients with portal hypertension could help identify individuals at higher risk of complications. Large-scale pharmacogenetic studies, encompassing genetic polymorphisms, are crucial for advancing personalized medicine in this field. It is also essential to develop standardized protocols for pharmacogenetic testing, which could guide therapy in clinical practice.

Given the complex pathophysiology of portal hypertension, an interdisciplinary approach is necessary. This requires the collaboration of pharmacologists, geneticists, and hepatologists to develop personalized therapeutic strategies that integrate genetic, molecular, and clinical data to achieve optimal treatment outcomes for patients with portal hypertension.

## 7. Pharmacogenetic Considerations in the Treatment of Portal Hypertension

Pharmacogenomics (PGx) aims to identify enhance therapeutic efficacy, and minimize adverse drug reactions [344]. Its objectives include correlating genetic variations with clinical phenotypes, enabling precise genotype-driven predictions of drug response and disease susceptibility, and utilizing pharmacogenetic profiling to optimize personalized medicine strategies [345]. Pharmacogenomics could play a crucial role in future in the management of portal hypertension by enabling personalized therapy to identify the most effective treatments for individual patients, optimizing drug selection based on genetic factors that affect liver function and drug metabolism, and minimizing adverse effects, particularly in patients with cirrhosis, where drug response may vary significantly [345].

CYP2C9, an enzyme within this system, is responsible for the oxidative metabolism of numerous drugs, including warfarin [346]. Additionally, irbesartan and losartan, which are also metabolized by CYP2C9, could potentially be utilized in the clinical treatment of portal hypertension [347]. Recent studies on warfarin dosing algorithms have shown that considering non-genetic factors such as age, sex, body mass index, diet, concomitant medications, ethnicity, food, and genetic variability in CYP2C9 and VKORC1 can significantly improve the accuracy of therapeutic decisions [348].

The allele frequencies of CYP2C9*2 and CYP2C9*3 vary significantly across populations, ranging from 4% to 16% in Caucasian populations [349], while CYP2C9*2 is absent in the Asian population, and the frequency of CYP2C9*3 ranges from 0.07% to 6.0% [350,351,352,353]. Patients with certain polymorphisms, like CYP2C9*2 or CYP2C9*3, may experience altered drug responses, including a higher risk of adverse effects or inadequate therapeutic efficacy [354]. Polymorphisms in the CYP2C9 gene can lead to reduced enzyme activity, resulting in slower metabolism of warfarin and consequently a higher risk of bleeding. Patients with these variants may require lower doses of warfarin to achieve optimal therapeutic effects and avoid serious side effects. This variability could contribute to the lack of response to standard therapies, making it essential to consider pharmacogenetic profiling when selecting treatment for patients with portal hypertension [354].

Several approved medications, including simvastatin (SLCO1B1) or clopidogrel (CYP2C19), are known to have pharmacogenomic relationships, where genetic variations can influence their efficacy and safety profiles [355,356]. Polymorphisms in the CYP2C19 gene are clinically relevant for clopidogrel, particularly in the prevention of thrombosis, including portal vein thrombosis. In patients with portal hypertension, impaired CYP2C19 activity may reduce the therapeutic efficacy of clopidogrel, thereby increasing the risk of thrombotic events. Furthermore, pharmacogenomic relationships play an important role, SLCO1B1 polymorphism has been shown to predict the risk of statin-induced myopathy, which enables more effective management of adverse drug reactions in patients prescribed statins [357]. Considering the potential increase in the area under the curve (AUC) of statins in patients with cirrhosis, identifying genetic predispositions to adverse events could significantly enhance patient selection and risk stratification, ultimately improving the safety and efficacy of statin therapy [358].

Similar genetic influences have been observed in response to carvedilol. A greater reduction in blood pressure was noted in individuals with the R389R genotype compared to those carrying the R389G variant [359]. Additionally, the S49R389/S49R389 haplotype has been identified as a potential predictor of therapeutic response, highlighting its utility in guiding personalized treatment strategies [360]. Leineweber et al. demonstrated in their study that patients with cirrhosis and portal hypertension exhibit distinct hemodynamic response patterns to propranolol, which are influenced by β2-adrenergic receptor gene polymorphisms [361]. Consistent with their hypothesis, individuals with the Gly16-Glu/Gln27 haplotypes showed an enhanced response to propranolol administration, reflected in greater reductions in hepatic blood flow, as well as increased systemic and hepatic sinusoidal resistance [361]. Conversely, the Arg16-Gln27 haplotype was associated with a diminished response to the drug. These findings underline the significance of pharmacogenetic factors in tailoring effective therapies for portal hypertension [361] and emphasize the necessity of considering genetic variability in therapy selection, which will enhance therapy efficacy and be a further step towards a personalized approach to PH treatment.

Despite the proven role of PGx in drug therapy, its implementation in clinical practice remains slow due to several limitations. Few hospitals or clinics globally integrate PGx tools into routine practice, with genetic testing not being routinely conducted for patients [362,363,364]. This is caused by several factors, including perceived insufficient clinical utility, a lack of cost-effectiveness studies, the absence of standardized genotyping tests for each drug-gene pair, challenges in interpreting PGx test results, and the lack of specific guidelines or a standardized protocol for managing patients with mutated pharmacogenetics [365].

Modern technologies, such as omics-based approaches and big data analytics, could allow future more personalized treatment strategies tailored to each patient’s unique genetic and clinical profile, which in turn can reduce the frequency of adverse drug reactions and improve the therapeutic efficacy of pharmacological treatments for portal hypertension [366].

Although there are currently no widely used pharmacogenetic tests in the treatment of portal hypertension PH, the development of this field could significantly contribute to the personalization of treatment in the future. The number of studies on genetic polymorphisms affecting drug response is steadily increasing, and it can be assumed that some FDA-approved tests will find practical clinical applications in PH therapy.

The Food and Drug Administration (FDA) has approved several test kits using microarray technology for CYP450 genes, such as AmpliChip CYP450, the first pharmacogenetic test approved by the FDA [367]. Another recent example is the Genomadix Cube CYP2C19 test, approved by the FDA in March 2023 [368]. This system provides results with 99.1% accuracy, allowing for the identification of CYP2C19 *2, *3, and *17 genotypes from a cheek swab sample [368]. By analyzing these genotypes, doctors can better tailor therapeutic strategies for drugs metabolized by the CYP450 2C19 enzyme within an hour [368], which may be particularly significant in the application of this test in the context of pharmacogenetic PH, given that CYP2C9 is mainly involved in the metabolism of Clopidogrel, Irbesartan, Losartan, and Sildenafil, which we currently use or will use in the future for the treatment of PH.

Such a personalized approach can improve treatment outcomes and reduce side effects for 10% of clinically used drugs [369,370]. Additionally, the FDA-approved xTAG CYP2D6 Kit V3 test detects genetic changes in the CYP2D6 gene, which metabolizes many drugs, including propranolol, commonly used in treating PH. This approach improves patient treatment outcomes and increases safety [368].

On the other hand, RT-PCR methods, such as TaqMan tests, use fluorescent probes to detect specific genetic variants [368]. In one study, RT-PCR with TaqMan probes specific to mutations was used to detect common allele variants in the CYP2C9, CYP2C19, and CYP2D6 genes [368]. Thanks to this precise detection of genetic variants, this technique has provided valuable data for personalized medicine approaches in the individual patient approach, especially since these isoenzymes metabolize drugs commonly used in PH therapy, such as warfarin, propranolol, and clopidogrel.

## 8. Conclusions

The management of PH remains a significant therapeutic challenge, particularly for patients who do not respond to or cannot tolerate NSBBs. Despite current first-line therapies, the need for more effective and well-tolerated treatments is urgent. Statins emerge as a promising option, offering potential benefits in reducing hepatic vascular resistance, mitigating fibrosis, and preventing severe complications like variceal bleeding and hepatic encephalopathy. Their survival benefit in early-stage cirrhosis (Child–Pugh A) and ongoing trials in advanced stages reinforce their therapeutic potential.

Several molecular pathways offer substantial therapeutic promise in PH management. Among these, the eNOS pathway holds the greatest promise due to its direct role in restoring endothelial function and improving intrahepatic circulation. Additionally, newer pharmacological agents such as AMPK activators and pan-caspase inhibitors may have the potential to reduce HVPG and improve liver function in the future, although further clinical research is needed to confirm these findings. These therapies, especially when targeting pathways involved in hepatic blood flow and fibrosis, could not only improve liver function but also reduce the risk of complications such as ascites and hepatic encephalopathy.

While these developments are encouraging, further studies are essential to solidify their role in standard PH therapy, and in managing its life-threatening complications. Future research should focus on synergistic strategies, such as combining statins with other agents, to enhance efficacy and reduce complications.

## Figures and Tables

**Table 1:** The main therapies used currently to treat portal hypertension.

Substance	Mechanism of Action	Approval Date	Indications	Pharmacokinetics
Propranolol	Beta-1 and beta-2 adrenergic receptors antagonist [130]	1967	Hypertension, angina, essential tremor, prevention of migraines, portal hypertension [157]	Propranolol has a half-life of approximately 3 to 6 h and is extensively metabolized by the liver via CYP2D6 [158]
Nadolol	Beta-1 and beta-2 adrenergic receptors antagonist [159]	1982	Hypertension, angina pectoris, portal hypertension management [160]	The half-life of nadolol is approximately 20 to 24 h. It is primarily excreted unchanged in the urine [161]
Terlipressin	Selective agonism of vasopressin V1 receptors [162]	1984	Acute variceal bleeding, hepatorenal syndrome, portal hypertension [163]	Terlipressin has a half-life of about 50 min and is primarily eliminated by the kidneys [151]
Octreotide	Somatostatin analog that inhibits the secretion of growth hormone, insulin, and glucagon [164]	1998	Acromegaly, neuroendocrine tumors, and variceal hemorrhage due to portal hypertension [164]	The half-life of octreotide is about 1.5 to 2 h, and it is primarily metabolized in the liver [165]
Carvedilol	Beta-1, beta-2, and alpha-1 adrenergic receptors antagonist [166]	1995	Hypertension and heart failure. Also used in patients with portal hypertension [167,168]	Carvedilol has a half-life of approximately 7 to 10 h and undergoes extensive hepatic metabolism [159]

**Table 2:** The currently pursued pharmacological interventions in portal hypertension (according to https://clinicaltrials.gov/ web-base, accessed on 10 October 2024).

Clinical Trail Phase	Mechanism of Action	Compound Names	Reference	Status
I	Selective vasopressin V2 receptor agonist	FE 204205	NCT02929407	Terminated
I	Selectively blocking the AT1	Fimasartan	NCT01146938	Completed
I	Activation of soluble guanylate cyclase (sGC)	BI 685509	NCT03842761	Completed
II	Activation of sGC	BI 685509	NCT05161481	Terminated
II	Activation of sGC	BI 685509	NCT05282121	Terminated
II	Activation of sGC	Avenciguat	NCT06082843	Terminated
II	Thromboxane A2/prostaglandin H2 receptor antagonist	Ifetroban	NCT02802228	Completed
II	Endothelin receptor antagonist (ETA)	Ambrisentan	NCT03827200	Terminated
II	ETA antagonist	Zibotentan	NCT05516498	Recruiting
II	Selective and potent inhibitor of the enzyme indoleamine 2,3-dioxygenase 1 (IDO1)	Emricasan (IDN-6556)	NCT02230683	Completed
II	Somatostatin analog	Sandostatin LAR	NCT01188733	Completed
II	Selective inhibitor of sodium-glucose cotransporter 2 (SGLT2)	Dapagliflozin	NCT05516498	Recruiting
III	Spasmolytic agent	Alverine	NCT06473493	Not recruiting yet
III	Spasmolytic agent	Alverine	NCT06470386	Not recruiting yet
III	NSBB and alpha-1 adrenergic receptor antagonist	Carvedilol	NCT00493480	Completed
III	Inhibits TNF-α, reducing inflammation	Thalidomide	NCT00787436	Withdrawn
III	Inhibiting HMG-CoA reductase	Simvastatin	NCT02134626	Completed
III	Galectin-3 inhibitor	Belapectin	NCT04365868	Active, not recruiting
IV	Osmolyte and antioxidant	Taurine	NCT02344719	Completed
IV	Inhibiting HMG-CoA reductase	Simvastatin	NCT02994485	Completed
IV	Potassium-sparing diuretic by antagonizing mineralocorticoid receptors	Spironolactone	NCT02907749	Completed
IV	PPAR-γ agonist	Pioglitazone	NCT00570622	Completed
IV	Selective agonism of vasopressin V1 receptors	Terlipressin	NCT00534677	Completed
IV	Selective agonism of vasopressin V1 receptors	Terlipressin	NCT02119884	Completed
IV	Synthetic analog of somatostatin	Octreotide	NCT00534677	Completed
IV	Synthetic analog of somatostatin	Octreotide	NCT02119884	Completed
IV	NSBB and alpha-1 adrenergic receptor antagonist	Carvedilol	NCT06449339	Recruiting
IV	NSBB and alpha-1 adrenergic receptor antagonist	Carvedilol	NCT01059396	Completed
IV	NSBB	Propranolol	NCT01059396	Completed

**Table 3:** Comparison of efficacy and limitations of traditional and new therapies for portal hypertension: clinical and experimental data.

Therapy	Mechanism of Action	Efficacy	Limitations
Current Therapies			
NSBBs	Decrease cardiac output and splanchnic blood flow [120,121]	Reduce portal pressure; prevent variceal bleeding [114]	Poor tolerance in 15% to 20% of patients; systemic hypotension; limited in decompensated cirrhosis [127,128,129,130,131]
Somatostatin analogs	Inhibit vasodilatory hormones to reduce portal venous inflow [164]	Effective for acute variceal bleeding; reduces early rebleeding rates [142]	Limited to acute conditions; primarily parenteral administration [143]
Vasopressin analogs	Selective agonism of vasopressin V1 receptors [162]	Effectively mitigates portal hypertension-induced ascites [144,145,147]	Short distribution half-life; necessitates continuous intravenous thereby restricting its use to acute care and hospital conditions; numerous adverse effects [151,153,154,155,156]
Emerging Therapies			
FXR agonists	Regulate bile acid metabolism; reduce fibrosis and vascular resistance [203,204,205]	Promising results in reducing portal pressure in animal models; clinical trials ongoing [222]	Limited human data; potential variation in response due to genetic polymorphisms in FXR pathways [67]
The amino acid taurine	Inhibit the activation of HSCs [227]	Pilot study showed a 12% reduction in HVPG after 4 weeks; well tolerated [194]	Limited clinical data; effects on long-term outcomes unknown
Mas receptor agonist	Enhance nitric oxide synthesis; attenuate Rho-kinase signaling [232]	Reduced portal pressure and vascular resistance in preclinical studies [232]	Early-stage development; lack of robust clinical trial data
Angiotensin II type 1 receptor blockers	Block angiotensin II type 1 receptors, reducing fibrogenesis and vascular resistance [231,235]	Reduced hepatic vascular resistance in compensated cirrhosis; no significant HVPG reduction in some trials [199,233]	Limited efficacy as monotherapy; potential for hypotension [235]
Endothelin-A receptor antagonists	Reduce intrahepatic vascular resistance [190]	Dose-dependent reduction in HVPG without systemic effects [236]	Limited trials; lack of significant efficacy in previous studies
Caspase inhibitors	Prevent hepatocyte apoptosis and reduce inflammation and fibrosis [201,237]	Emricasan reduced HVPG in patients with baseline ≥ 12 mmHg; improved liver function markers [202,238]	Mixed results in trials; further research needed to confirm benefits
Statins	Inhibit HMG-CoA reductase; increase NO bioavailability [230,242,243]	Reduce portal pressure; antifibrotic effects; lower risk of decompensation; current data suggest potential benefits [246,247,248]	Risk of rhabdomyolysis in advanced cirrhosis; contraindicated in some patients with advanced liver dysfunction [252,253]
PCSK9 inhibitors	Increase LDL receptor availability; modulate endothelial dysfunction [258,259,260]	Potential benefits for lipid metabolism and vascular dynamics; synergistic with statins [261,262,263]	Minimal direct evidence in PH; requires large clinical trials, costly
PDE5 inhibitors	Prolong NO-cGMP signaling by inhibiting cGMP degradation [266]	Decreased HVPG in animal models; udenafil showed a ~20% reduction in portal pressure [267,268]	Contradictory results; potential systemic side effects, including renal function deterioration [268,269]
NOX inhibitors	Reduce oxidative stress by targeting NADPH oxidases [289,290]	Preclinical data suggest potential benefits in reducing fibrosis and portal pressure [290,292]	Lack of clinical trial data; unclear therapeutic window
Rho-Kinase Inhibitors	Modulate vascular tone by inhibiting Rho-associated kinases [308]	Preclinical studies demonstrated reduced hepatic vascular resistance and portal pressure [309,310]	No significant clinical trial data available; safety profile unverified [309,310]
Metformin	Activates AMPK, reducing hepatic inflammation and improving insulin sensitivity [315,316,317]	Preclinical studies show antifibrotic effects [316]	Limited human data; contraindicated in advanced liver disease due to lactic acidosis risk

## Data Availability

No new data were created or analyzed in this study.
